# NANOG Dominates Interleukin-6-Induced Sphere Formation in Prostate Cancer

**DOI:** 10.5152/tud.2023.23116

**Published:** 2023-11-01

**Authors:** Didem Seven, Didem Tecimel, Ömer Faruk Bayrak

**Affiliations:** Department of Medical Genetics, Yeditepe University School of Medicine, Istanbul, Turkey

**Keywords:** Prostate cancer, tumorsphere, IL-6, NANOG gene

## Abstract

**Objective:**

Identifying the dynamics of prostate tumor aggressiveness is essential to find new therapeutics for the treatment. Cancer stem cells contribute to cancer progression by promoting tumor growth and metastasis, resisting treatment, and evading the immune system. Interleukin 6 (IL-6) is a pleiotropic cytokine that functions in inflammation, immune response, etc. However, dysregulated expression of IL-6 plays a pathological role in such conditions as cancer. In this study, we aimed to elucidate the effect of IL-6 on cancer stemness genes in prostate cancer cells.

**Methods:**

Enrichment of stem-like cells was achieved through the formation of tumor spheres using the DU-145 cell line. Sphere formation was conducted in a medium supplemented with IL-6 and compared to a control group. The number of spheres was quantified, and the resulting pellet was collected for quantitative reverse transcription polymerase chain reaction analysis to assess the impact of IL-6 induction on the expression of stemness-related genes.

**Results:**

Tumor sphere numbers and sizes increased in IL-6-induced environment. NANOG expression elevated in an IL-6-enriched environment compared to the non-treated spheres. Our results demonstrated that IL-6 induction in prostate tumor spheres upregulates NANOG gene expression.

**Conclusion:**

Inducing IL-6 in prostate tumor spheres stimulates stemness biomarker NANOG genes. NANOG may be suggested as a therapeutic target for metastatic prostate cancer.

**Main Points**

- Elevated levels of interleukin 6 (IL-6) in tumor microenvironment are associated with tumor growth, invasiveness, and metastasis.
- Cancer stem cells can be mimicked by using tumorspheres.
- A small number of single prostate cells are adequate to can form tumor spheres in an environment where IL-6 is present. These tumor spheres are also larger than those formed in an environment without IL-6.
- The expression of stemness biomarkers is upregulated in prostate cancer spheres when they are exposed to IL-6.
- The promotion of stemness in prostate tumorspheres by the addition of IL-6 cytokine is driven by an increase in NANOG gene expression.

## Introduction

Prostate cancer (PCa) is the fourth most common cancer worldwide and one of the main causes of cancer-related deaths in males. An estimated 1.5 million people are diagnosed with PCa and approximately a quarter of it results in death.^1^ Prostate cancer is observed in men aged after 50, peaking between the ages of 75 and 79.^2^ In addition to age, family history, race, obesity, and environmental factors are other risk factors for PCa.^3^ About 5% of men diagnosed with PCa have distant metastases, often to multiple sites like brain, lungs and liver. Another 15% of men have locoregional metastases, which means the cancer has spread to nearby lymph nodes. Men with distant metastases have a poor overall survival rate of only 30% for 5 years. High rates of relapse, metastasis, and drug resistance continue to be a significant concern despite the fact that modern screening programs have enabled earlier identification and treatment.^4^ The background of these concerns in PCa should be clarified to find new therapeutic targets.

In several malignancies, including PCa, a small subpopulation of malignant cells exhibiting stemness characteristics have been found and defined as cancer stem cells (CSCs).^5^ Cancer stem cells, a small cell fraction inside the tumor that is assumed to be responsible for heterogeneity, resistance, recurrence, and metastasis of cancers, are characterized by self-renewal, differentiation, long-term culture, and drug resistance potential.^6^ There is growing evidence that CSCs are regulated by *Oct4, Sox2, Klf4*, and *Myc* (OSKM) genes that are called Yamanaka factors.^7^ The other important collaborator of OSKM genes in pluripotency and stemness is the *NANOG *gene, which is an essential transcription factor for maintaining pluripotency in embryonic stem cells. Aberrant expression of the Yamanaka factors and NANOG gene was shown in glioblastoma, breast, lung, colorectal, pancreatic, ovarian cancers, and PCa.^8-10^ *NANOG* is overexpressed in CSCs, and its inhibition can lead to the death of stem cell-enriched population; thus, it is suggested as a therapeutic target.^11^

Interleukin 6 (IL-6), one of the important cytokines, is involved in the regulation of cellular stemness by increasing transcriptional factors.^12^ Interleukin 6 levels are normally quite low in the typical homeostatic condition, whereas a wide range of cells release IL-6 in response to abnormalities such as inflammation. Interleukin 6 is a main component that is ubiquitously found and known to be dysregulated in cancer. Various PCa research studies have associated IL-6 increment with tumor aggressiveness. Most of the PCa cells show elevated levels of IL-6 and exhibit malignant potential.^13^

Overexpression of IL-6 is associated with tumor aggressiveness in PCa. The majority of PCa cell lines express IL-6; especially, the cell lines that are devoid of androgen receptor exhibit increased malignant potential due to elevated levels of IL-6.^14^ In this study, we aimed to investigate the IL-6-induced stemness properties of PCa cells.

## Material and Methods

### Cell Culture

The DU-145 PCa cells were obtained from American Type Culture Collection (Manasas, Va, USA). Considering the cells were provided commercially, no ethical approval is required for this study. The cells were cultured in RPMI1640 Medium (GIBCO, Grand Island, NY, USA) medium with 10% FBS (GIBCO, USA) and 1% penicillin–streptomycin (GIBCO, USA) in a humidified incubator maintained at 37°C with 5% CO_2_ in air. The cells were shown to be mycoplasma free. When they reached 80% confluency, the cells were trypsinized. The medium was changed 3 times a week.

### Sphere Formation

A total of 30 000 DU-145 single cells were seeded in RPMI medium containing EGF (10 ng/mL) (GIBCO, USA) and bFGF (10 ng/mL) (GIBCO, USA), 2% B27 (GIBCO, USA), 1% N2 (GIBCO, USA), and 1% penicillin–streptomycin in ultra low-attachment 6-well plate (Corning Inc., Corning, NY, USA). To assess the sphere-forming potential of a population of cells, between 500 and 2000 cells were seeded in each well of a 24-well plate. Sphere medium was added on the third day and was changed once in a week. Spheres were counted after 10 days. Human recombinant IL-6 protein (Gibco, Thermofisher Scientific) at a concentration of 10 ng/µL was added to the sphere medium to detect IL-6-induced effect. The microscopic images were recorded for each day and the experiments repeated in triplicate.

### Ribonucleic Acid Extraction and Reverse Transcription

Spheres were collected from 6-well plates on day 10 for RNA extraction. The media was collected in a centrifuge tube and spin down for 5 minutes at 1200 rpm. The supernatant was removed, and the pellet was stored for RNA extraction. Total RNA was isolated from each sphere pellets by using TRIzol Reagent (Invitrogen Life Technologies, Carlsbad, Calif, USA). The purity and yield of RNA were measured by using NanoDrop ND-1000 spectrophotometer. 1000ng of total RNA was reverse-transcribed into cDNA in 20 μL final volume using the High-Capacity cDNA Reverse Transcription Kit (Applied Biosystems, Rotkreuz, Switzerland).

### Real-Time Polymerase Chain Reaction Experiment

Quantitative reverse transcription polymerase chain reaction was performed by using a StepOnePlus instrument (Applied Biosystems, Foster City, Calif, USA). TaqMan gene expression assays were used to detect OCT4 (Hs04260367_gH), SOX2 (Hs04234836_s1), KLF4 (Hs00358836_m1), and c-MYC (Hs00153408_m1) genes. Polymerase chain reaction (PCR) was set up by mixing 0.5 µL TaqMan Assay, 5 µL TaqMan Universal Master Mix (Applied Biosystems, Rotkreuz, Switzerland), 50 ng of cDNA, and the final volume was completed to 10 µL with 2.5 μL of PCR-grade water. Amplification and real-time data acquisition were run using the following cycle conditions: 10 minutes at 95°C, followed by 40 cycles of 15 seconds at 95°C, and 1 minute at 60°C. The threshold cycle values was used to evaluate the fold change. The expressions of genes were normalized against those of housekeeping genes *YWHAZ* (Hs01122445_g1) and *TBP* (Hs00427620_m1). The 2^−∆∆CT^ method was used to calculate fold change.^15^ The experiments were carried out in triplicate.

### Statistical Analysis

Graphpad Prism 8.4.3 (GraphPad Software, Inc. CA, US) was used to analyze the data. *t*-test was applied to compare sphere formation and Wilcoxon rank test was used to analyze fold change of the genes. A *P*-value less than .05 value was considered significant.

## Results

**Figure 1.**
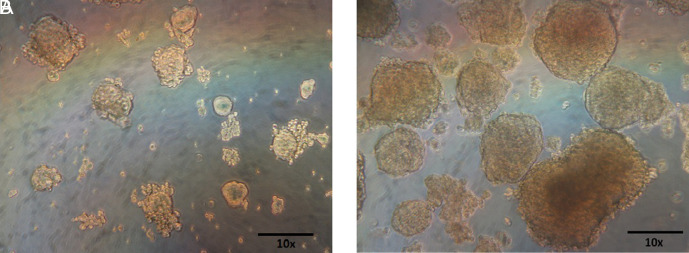
Prostate tumorspheres. (A) DU-145 tumorspheres. (B) Interleukin 6-induced DU-145 tumorspheres.

**Figure 2.**
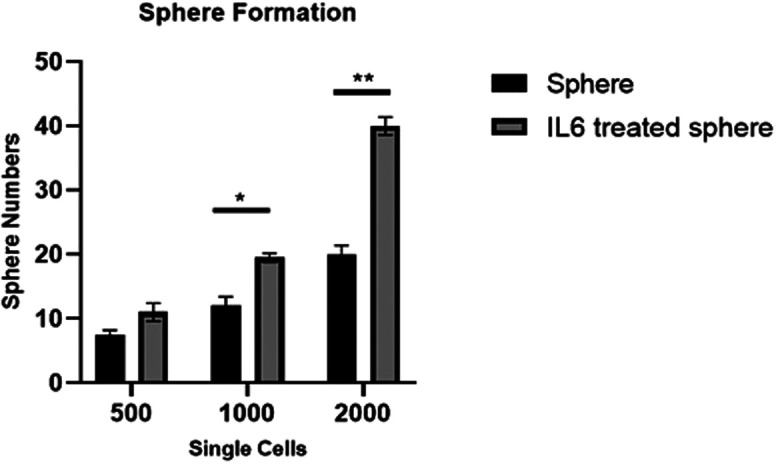
Sphere formation assay.

We observed DU-145 sphere formation under light microscopy. Both DU-145 spheres and IL-6-induced prostate tumor spheres were successfully generated. In addition, the size of the spheres was larger in the IL-6-treated group (Figure 1). To check the self-renewal capacity of the spheres, we seeded different amounts of single cells in IL-6-treated medium and counted the spheres after 10 days. We showed that PCa cells could generate at least 10 spheres when we initiated with 500 K cells. Starting with 1,000 to 2,000 cells resulted in a significant increase in the number of tumor spheres formed. The addition of IL-6 to the medium with an initial cell population of 1000 cells yielded approximately 20 spheres, whereas the increase in cell count to 2000 cells corresponded to doubling in the number of spheres formed. A notable upregulation was observed from the initial cell count of 1000 cells onward (Figure 2). 

**Figure 3.**
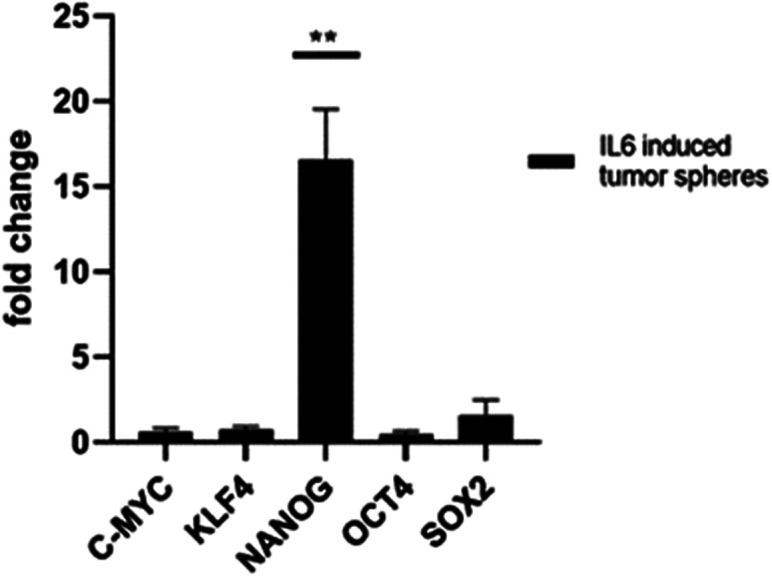
Fold change of the genes in interleukin 6-induced tumor spheres.

To determine whether IL-6 has an effect on PCa stemness, we collected both tumor spheres and IL-6-treated sphere pellets. OSKM and *NANOG *gene expression levels were measured. Despite a slight upregulation detected in the *SOX2* gene, the alteration was not significant. The expression of *KLF4, OCT4, and MYC* genes was not changed. However, a dramatic increase was observed in NANOG expression on IL-6-treated PCa spheres. A representative image of the fold changes of the genes is shown in Figure 3. 

## Discussion

The aggressive environment of metastatic PCa was mimicked by adding IL-6 to tumor spheres in this study. We observed that IL-6 promoted cancer stemness, as evidenced by the dramatic increase in NANOG expression.

The 5-year survival rate of metastatic PCa is roughly 30%, despite treatment options such as radiation therapy, chemotherapy, hormone reagents for hormone-sensitive PCa, or combination therapies. Understanding the structure of aggressiveness is important to find new and functional therapeutics.

Cancer stem cells are known to have the ability to differentiate and self-renew. Moreover, they are thought to play a role in cancer relapse, metastasis, and drug resistance.^16^ Tumor spheres are 3-dimensional models of CSCs/progenitor cells that are generated from small groups of cancer cells. These stem-like structures ensure to figure out the cancer aggressiveness.^17^ Tumorsphere are believed to play a critical role in prostate tumor initiation, progression, and resistance to therapy. In addition, Tumorspheres are thought to better recapitulate the properties of CSCs than traditional 2-dimensional cultures.^18^ In our study, we obtain tumor spheres by initiating a low amount of cells, which supports the idea that each sphere originates from a single progenitor cell.^19^ Morever, the addition of IL-6 to the sphere-forming media has resulted in larger, and increased number of spheres, which is thought to cause a more aggressive phenotype. IL-6 is a pro-inflammatory cytokine and acts as a signaling molecule in tumorigenicity. It is well known that both tumor tissue and PCa patients serum have high levels of IL-6.^20^ It is also demonstrated that IL-6 stimulation boosts PCa proliferation assistant with signal transducer and activator of transcription 3 (STAT3)-activated signaling pathway.^21^ Furthermore, it has a role in regulating stemness, and studies showed that siltuximab treatment, which inhibits STAT3, suppressed the clonogenicity of PCa stem cells.^13,22^

In the PCa microenvironment, the pleiotropic IL-6 expression could be the result of any dysregulated mechanisms. Yu et al^23^ studied the effect of IL-6 on PC-3 and lymph node carcinoma of the prostate (LNCaP) PCa cells and observed the *SOX2* increment. Drug resistance effect of IL6 on stromal fibroblast was studied by Niu N et al,^24^ and stemness properties in an IL-6 enriched sphere environment was investigated. According to their results, *NANOG* and *SOX2* were the most affected genes from IL-6 addition. Even though there was a minor increment in *SOX2 *expression in our IL-6-induced prostate tumorspheres, it was not significant. When we checked the stemness properties in an IL-6-induced environment, the expression of *OCT4, KLF4, *and c-Myc did not change. However, we detected a serious upregulation in *NANOG* expression. The convincing evidence manifested that *NANOG* has a crucial role in self-renewal and differentiation of stem cells, and it could be responsible for the oncogenic process.^25^ Bao B et al^26^ previously showed that PCa cells, PC3 and LnCap, increase the level of IL-6 and vascular endothelial growth factor in hypoxic conditions that affect CSCs and increase the level *of NANOG *gene. Shroeder et al^27^ supported our data by establishing that loss of IL-6 caused the inhibition of CSC population and diminished NANOG protein expression. As IL-6 expression upregulate in most of the pathologic conditions, not only hypoxia but also autoimmune and inflammatory situation, we directly induced IL-6 and obtained the results as Bao B et al^28^ and confirmed the Shroeder et al^29^ results. The research proved that *NANOG* has a key role in the development of castration-resistant PCa and could be a promising target for the advanced stage of PCa. Considering the significant alteration in gene expression observed only in the *NANOG* gene among the embryonic stemness markers, it can be assumed that IL-6-induced stemness could be dominated by *NANOG*. Recently, it has been demonstrated that there is a strong link between *NANOG *gene and IL-6 signaling in esophageal squamous carcinoma, as *NANOG* is one of the downstream regulators of STAT3.^30^ Therefore, we think that the same mechanism could be responsible for PCa stemness, as *NANOG* promotes the formation of PCa stem cells in an IL-6-rich environment that can be considered as a crucial factor for tumor aggressiveness. Ongoing animal experiments using siNanog or shNanog have shown the promising therapeutic potential of NANOG targeting in several types of cancer. Additionally, NANOG inhibitors BBI608 or BBI503 were combined with sorafenib in adult patients with hepatocellular carcinoma in a phase II study (NCT02279719).^31^ Promising antitumor effects have been observed in patients with hepatocellular carcinoma who have not undergone systemic chemotherapy previously, and the trial was completed in 2002.

The limitation of this study is that the levels of NANOG gene expression was measured in only one type of PCa cell line. In future studies, it is necessary to measure the protein levels of NANOG in an IL-6-rich environment.

In this study, *NANOG* is shown to have an important role in the stemness of PCa in an IL-6-enriched environment. Understanding the role of NANOG in CSCs has implications for developing targeted therapies and improving cancer treatment outcomes.
